# Historical navigation routes in European waters leave their footprint on the contemporary seascape genetics of a colonial urochordate

**DOI:** 10.1038/s41598-023-46174-0

**Published:** 2023-11-04

**Authors:** Eitan Reem, Jacob Douek, Baruch Rinkevich

**Affiliations:** Israel Oceanography and Limnological Research, National Institute of Oceanography, Tel Shikmona, P.O. Box 9753, 3109701 Haifa, Israel

**Keywords:** Ecology, Evolution, Genetics

## Abstract

Humans have intensively sailed the Mediterranean and European Atlantic waters throughout history, from the upper Paleolithic until today and centuries of human seafaring have established complex coastal and cross-seas navigation networks. Historical literature revealed three major long-lasting maritime routes (eastern, western, northern) with four commencing locations (Alexandria, Venice, Genoa, Gibraltar) and a fourth route (circum-Italian) that connected between them. Due to oceangoing and technological constraints, most voyages were coastal, lasted weeks to months, with extended resting periods, allowing the development of fouling organisms on ship hulls. One of the abiding travellers in maritime routes is the colonial ascidian *Botryllus schlosseri* already known since the eighteenth century in European and Mediterranean ports. This species, was almost certainly one of the common hull fouling travellers in all trade routes for centuries. Employing *COI* haplotypes (1008 samples) and microsatellite alleles (995 samples) on colonies sampled from 64 pan-European sites, present-day *Botryllus* populations in the Mediterranean Sea/European Atlantic revealed significant segregation between all four maritime routes with a conspicuous partition of the northern route. These results reveal that past anthropogenic transports of sedentary marine species throughout millennia long seafaring have left their footprint on contemporary seascape genetics of marine organisms.

## Introduction

**Figure 1:**
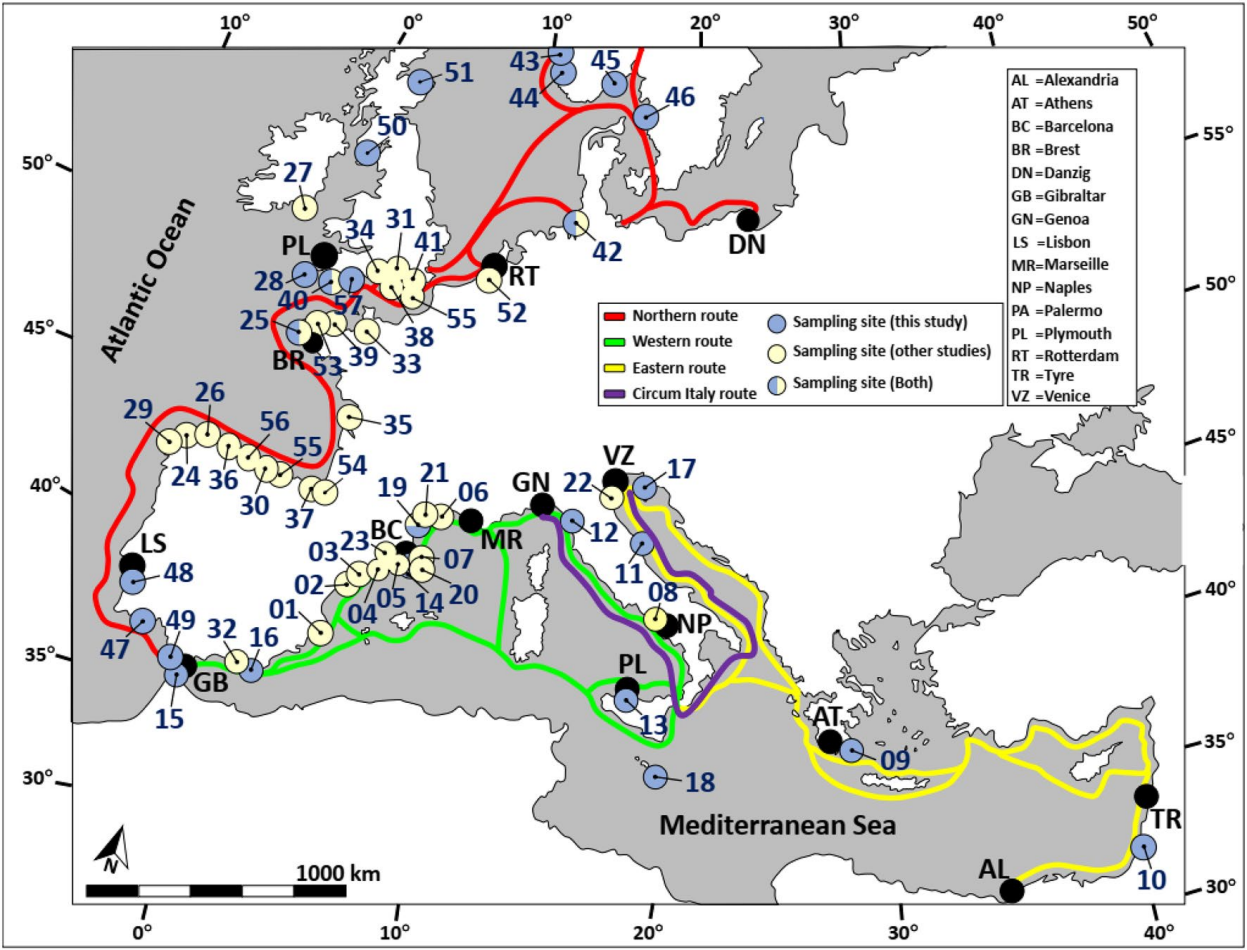
Mediterranean-sea and western European major trade routes, primarily along the coasts (sensu^14,62^). Black circles: main ports. Each number represents a sampling site. Sites identifications in Appendix Table 1.

It is not really known when humans began cross sailing the Mediterranean Sea and European Atlantic, albeit the availability of documentation from the upper Paleolithic period from about 11,000 BCE^1^, and crossing the English Channel, as well as from the Bronze Age, about 1500 BCE^2,3^. Yet, it was only in the ninth century BCE that the “Phoenician commerce across the Mediterranean and beyond had taken off”^1^ with movement from Tyre, Cyprus, Rhodes, Crete, the Ionian Sea to Sicily, the Balearic Islands, the Straits of Gibraltar to Cadiz and even further north. More or less during the same period, the Greeks crossed the Ionian Sea and travelled to Italy^1^ and further west through the Straits of Gibraltar towards Atlantic Europe^4,5^. The traffic in the Mediterranean was augmented during the Roman Empire by thousands of ships that sailed from “anywhere to everywhere”, carrying merchandises^6–8^, people and troops. this was followed by bustling maritime traffic during the Byzantine and Middle-ages, governed by the port-hubs of Venice, Genoa and Pisa alongside with the Mamluk and Ottoman Empires^1^. The sea going ties between Western Europe (Atlantic coasts) and the Mediterranean intensified during the Viking age (793–1066 CE) when the Norse people of Scandinavia explored, traded with and raided Britain, Iberia, southern coasts of France and northern Italy^9,10^. Starting from the thirteenth century, the Italian state cities of Genoa and Venice established extensive maritime trading relations with Northern Europe^1,11,12^. Thus, centuries of trade routes have established complex networks of European navigation courses with prominent trajectories along the coasts and cross-seas (Fig. 1).

Due to oceanographic constraints (waves, currents, winds, storms), readiness of navigation tools, ship design, oars and sail usage, most ships travelled for centuries along the same sea routes and sailing was almost entirely coastal (Fig. 1). As a result, en route trips lasted for weeks to months, relying on crews’ experience and land identifiable topography^1,7,13,14^. These slow voyages were repeatedly and frequently halted for water and food refills, resting and long mooring periods, creating ideal conditions for the accumulation of fouling marine organisms and their perpetuation on submerged surfaces. Slow sailing also contributed to this phenomenon. Hull fouling is known from ancient times, already marked by the Phoenicians, the Greeks and the Romans, and centuries thereafter^15,16^.

One of the notorious invasive species of the hull fouling consortia is the colonial ascidian *Botryllus schlosseri*^17–20^, a common species in marinas and ports worldwide, easily spotted on submerged manmade objects like ropes, buoys and floating docks. Besides being found on boat hulls, *Botryllus schlosseri’s* spreading mode doesn't exhibit any sort of distance-based isolation, thus suggesting that its distribution is likely a result of human-mediated dispersal, primarily facilitated by the ongoing movement of vessels fouled by sedentary marine organisms^21^. This species, most likely of a European or Mediterranean Sea origin^22,23^, was almost certainly one of the common hull fouling travelers in all trade routes for centuries. It is thus implied that the interminable trafficking in the Mediterranean Sea and European Atlantic maritime routes may have carried its footprint forward towards the current seascape genetics of this species, reflecting long-term balance between immigration, extinction and diversification. Hence, past human-mediated dispersal activities may reveal current “historical inference from genetic data”^24^. Studying the present-day *Botryllus schlosseri* populations in the Mediterranean Sea and European Atlantic^21,23,25–30^, we searched here for past maritime routes’ footmarks on current portraits of *COI* haplotypes and microsatellite alleles repertoires.

## Materials and methods

### *COI*—animal sampling and DNA extraction

Samples were collected from *Botryllus schlosseri* colonies residing in 23 Mediterranean Sea and Atlantic European sites (Fig. 1; Appendix Table 1), from the underside of stones or floating docks, submerged ropes and buoys in marinas, usually 0.1–0.5 m below sea level. Sampling was restricted to specimen > 1 m away from each other’s, to avoid kin colonies^30,31^ or ramets of the same genotype. Tissue samples were removed from the substrates using single-edge razor blades, and placed, individually, in 1.5 ml vials containing 240 µl of lysis buffer (0.25 M Trisborat pH 8.2, 0.1 M EDTA, 2% SDS, 0.1 M NaCl and 0.5 M NaClO_4_), homogenized, and equal volumes of Phenol/Chloroform/isoamyl alcohol (25:24:1) were added through mixing. The vials were shipped to the laboratory at the National Institute of Oceanography, Haifa, Israel and kept at 4 °C until used. Genomic DNA was extracted according to^30,32^, quality was evaluated using gel electrophoresis and nanodrop, and samples were kept as stock at 4 °C.

### COI amplification and analyses

DNA dilutions (1:50 or 1:100) were used in sterile double distilled water. A partial sequence of ~ 700 bp of the *COI* gene was amplified, using the marine invertebrates *COI* universal primers (HCO2198r, 5′TAAACTTCAGGG TGACCAAAAAATCA 3′ and LCO1490f., 5′GGTCAACAAATCATAAAGATATTGG 3′^33^). The PCR reaction was performed in 50 µl REDTaq Readymix solution (Sigma), containing 0.5 µM of each primer and 100–500 ng of template DNA. A single soak at 94 °C for 2 min was followed by 40 cycles of 94 °C for 1 min, 50 °C for 1 min and 72 °C for 1 min 30 s and a final extension step at 72 °C for 10 min. PCR products were screened on 1.25% agarose gel and successful products were sent for sequencing (Macrogen Inc., South Korea). BioEdit^34^ and ClustalX^35^) packages were used for sequence analyses and multiple alignments of the *COI* sequences.

### GenBank *COI* entries

GenBank deposited *COI* entries pertaining to additional 33 Mediterranean and East Atlantic populations were mined (Appendix Table 1), including the information for sampling sites, articles details and authors.

### Construction of putative maritime routes

Historical maritime data for shipping intensity, directions and main ports across the Mediterranean Sea and European Atlantic coasts as well as oceanographic information (winds directions and sea currents), covering > 3000 years, from the second millennium BCE until the eighteenth century CE, were mined from the literature. This maritime data, and primarily selected overviewing 16 historical sources (books, articles and websites; Appendix Text) and additional 10 sources (list of reference), enabled us to construct major pan European putative maritime routs.

### COI Data analyses

Data analysis was performed by implementing four consecutive steps: (1) Fisher Exact test and Cramer’s V effect size test examined the differences between the maritime routes using SPSS-23 software; (2) genetic distances, population structures and differences between maritime routes, including principal coordinate analysis (PcoA), were employed by using GenAlEx version 6.5 software^36^; (3) genetic population structures (no prior assumption) were performed with NetStruct^37^), to construct inter-individual genetic similarity networks, clustering them into groups of genetically similar individuals; (4) cluster and geneflow analyses were performed with BAPS software^38^).

### Microsatellites data analysis

Data pertaining to the distribution of four microsatellites in 32 sampled sites along the coasts of Scandinavia, western Europe, England and the Mediterranean^21,23,25,29^; Fig. 1) corresponded almost perfectly to the four chosen historical shipping routes of this study. The data was re-analyzed, using SPSS-23 software for Fisher Exact test and Cramer’s V effect size test, and GeneAlEx 6.5 was employed for the principal coordinate analysis, Fst, Nei genetic distance and Dest. Netstruct and BAPS software were used for cluster analysis and Geneflow.

## Results

### Historical shipping routes

The historical maritime data for shipping intensity and directions across the Mediterranean Sea and European Atlantic coasts^1,4,5,7^; Appendix Text) revealed that: (1) The Phoenicians sailed in the Mediterranean eastern basin starting in the fifteenth century BCE, and gradually moved westward, along the North African and European coasts towards Iberia^8^); (2) Starting from the first millennium BCE, the Greeks sailed in the same Phoenician routes, at first in the eastern basin and later, along European coasts towards Italy, France and Iberia; (3) Very intensive maritime traffic during the Roman Empire (27 BCE-476 CE), with ships sailing in all directions, and intensive cabotage by small vessels^7^. In addition, brisk traffic was also found in the eastern basin (e.g., 140–150 ships/year from Alexandria to Rome carrying wheat and barley grains^6,8^; Appendix text 7); 4. At the second half of the thirteenth century CE, Italian vessels (primarily from Genoa) crossed the straits of Gibraltar towards the British Iles, Scandinavia and the Baltics. Simultaneously, Venice dominated the eastward lanes; 5. During the fifteenth and sixteenth centuries, there was a substantial increase in traffic intensity (mainly cargo vessels) between the Italian city states of Genoa, Pisa and Venice and Atlantic European states in the North Sea, the Baltic states and Sweden; 6. During the eighteenth century, an intensive maritime trade existed between Marseilles and Atlantic Europe (averaging 183 ships/year^39^; Appendix text 6).

The above and additional detailed literature (Appendix Text) enabled us to draw three putative Mediterranean/Atlantic Europe major maritime routes (and three starting reference sites) and a fourth route that connected between two major routes (Fig. 1), as follows: (1) Eastern route-the port of Venice as the reference starting point for eastern Mediterranean trajectories, with 6 posteriorly *COI* sampling sites and 5 microsatellites sampling sites in Sicily, the Adriatic, Greece and the Levant; (2) Western route-the port of Genoa as the reference starting point for western Mediterranean trajectories, including the ports of Sicily, Rome, Marseilles, Barcelona and Gibraltar with 18 posteriorly *COI* sampling sites and 7 microsatellites sampling sites; (3) Northern route-the port of Gibraltar as the reference starting point inclusive 33 posteriorly *COI* sampling sites and 21 microsatellites sampling sites along the Atlantic-Iberian coasts, the English-channel, Ireland, Scotland, Holland, Germany and Scandinavia; (4) Circum-Italian route-connecting the eastern and western Mediterranean routes, revealing shipping trajectories that hugged the Italian peninsula between Venice and Genoa with vessels trafficking both westward and eastward and back since the Roman times (six *COI* sampling sites and four microsatellites sampling sites). Thus, some of the Italian sites included in eastern or western routes were also included in the circum-Italian route (Fig. 1; Appendix Table 1).

### *COI* analyses

*COI* sequences from 1008 *Botryllus schlosseri* samples (419 this study, 589 GenBank deposited, alignment length: 473 bp) were assigned to 57 sites in three marine/oceanic regions: East Mediterranean, West Mediterranean and Atlantic Europe (including Spain, France, England, Ireland, Holland, Germany, Scotland, Scandinavia) along the four given routes (Table 1; Appendix Table 1). Results revealed 101 *COI* haplotypes. As singletons (66; appeared just once in a single sampling site) and route-private haplotypes (24; appeared in only a single route) might have biased conclusions by reinforcing route disparity through local mutations or irregular connectivity episodes. Therefore they were excluded from the analyses which resulted in 11 haplotypes (Accession numbers are found in Appendix Table 2) that appeared in at least two out of the four maritime routes and a total of 818 specimens (Table 1a, b). A second outcome was the highly diverse distributions of the *COI* haplotypes among the oceanic regions (Table 1b).

**Table 1:** *COI* analyses. (a) Sampling sites, maritime routes and haplotypes in the Mediterranean Sea and European Atlantic regions. The selected *COI* haplotypes appear in more than a single route. (b) Distribution of each of the 11 selected *COI* haplotypes in each of the four maritime routes. In parentheses, percentages from each route samples. *Shared samples/haplotypes with the eastern and western Mediterranean route.

Marine/oceanic region	Sampling sites (n)	Samples(n)	*COI* haplotypes (n)		
(a)					
East Mediterranean (Eastern)	6	125	9		
West Mediterranean (Western)	15	278	5		
European Atlantic (Northern)	31	330	11		
Circum-Italian*	5*	85*	2*		

Haplotype code	Eastern route	Circum Italian route	Western route	Northern route	Total
(b)					
4	48 (38%)	78 (92%)	201 (72%)	48 (15%)	375
5	7 (6%)	0	43 (15%)	19 (6%)	69
6	0 (0%)	0	3 (1%)	31 (9%)	34
7	0 (0%)	0	23 (8%)	4 (1%)	27
9	4 (3%)	0	8 (3%)	143 (43%)	155
22	50 (40%)	0	0	1 (0.3%)	51
24	4 (3%)	0	0	48 (15%)	52
25	3 (2%)	0	0	2 (1%)	5
30	1 (1%)	0	0	10 (3%)	11
67	1 (1%)	0	0	6 (2%)	7
71	7 (6%)	7 (8%)	0	18 (5%)	32
Total	125	85	278	330	818

**Figure 2:**
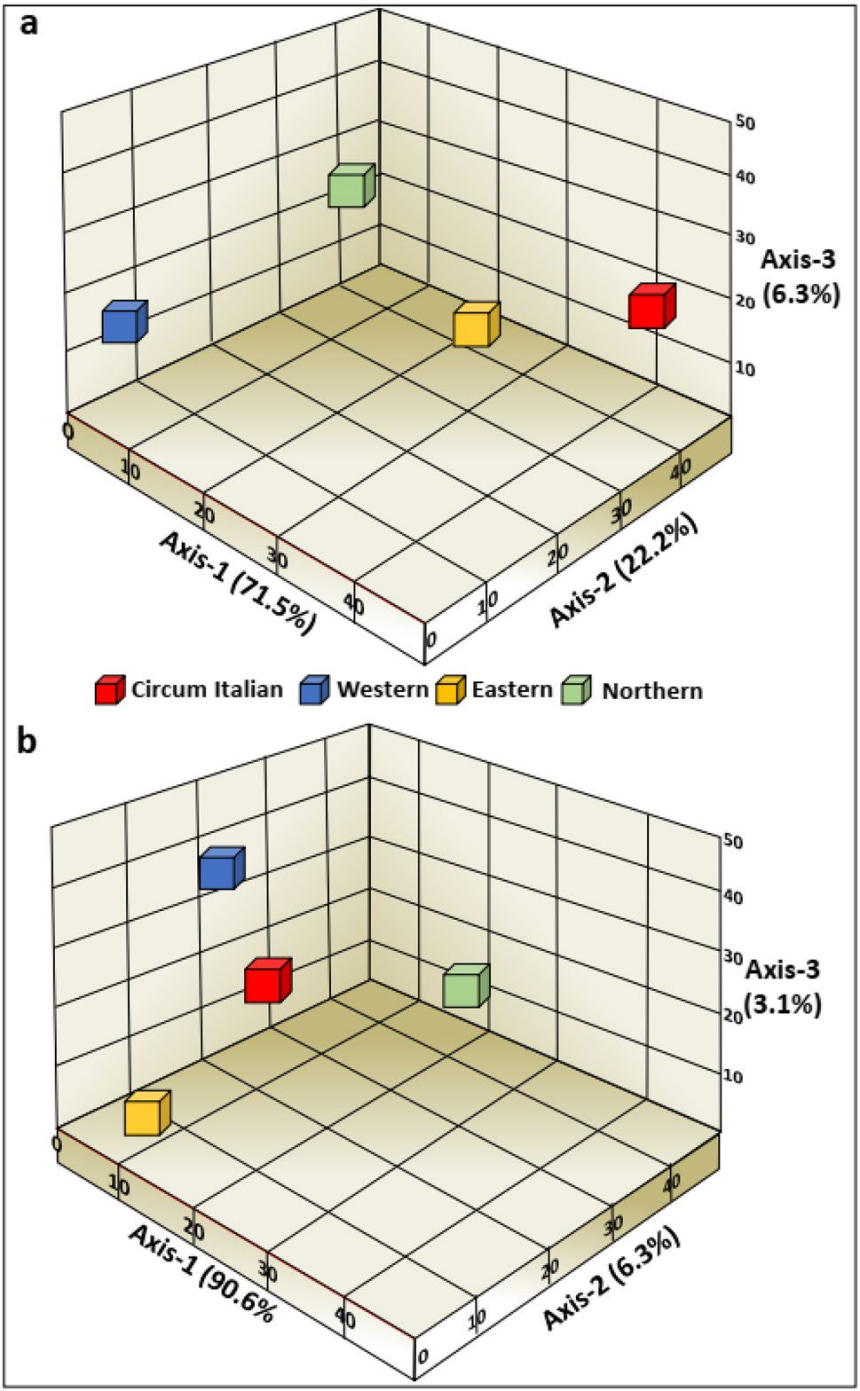
Principal coordinate analyses of the four maritime trade routes based on pairwise Nei genetic distance. (**a**) *COI*, revealing cumulative 93.7% of the explained variation in axes 1 and 2; (**b**) microsatellite alleles, revealing cumulative 96.9% of the explained variation in axes 1 and 2. Each of the four coloured cubes represents one of the major sailing trajectories.

**Figure 3:**
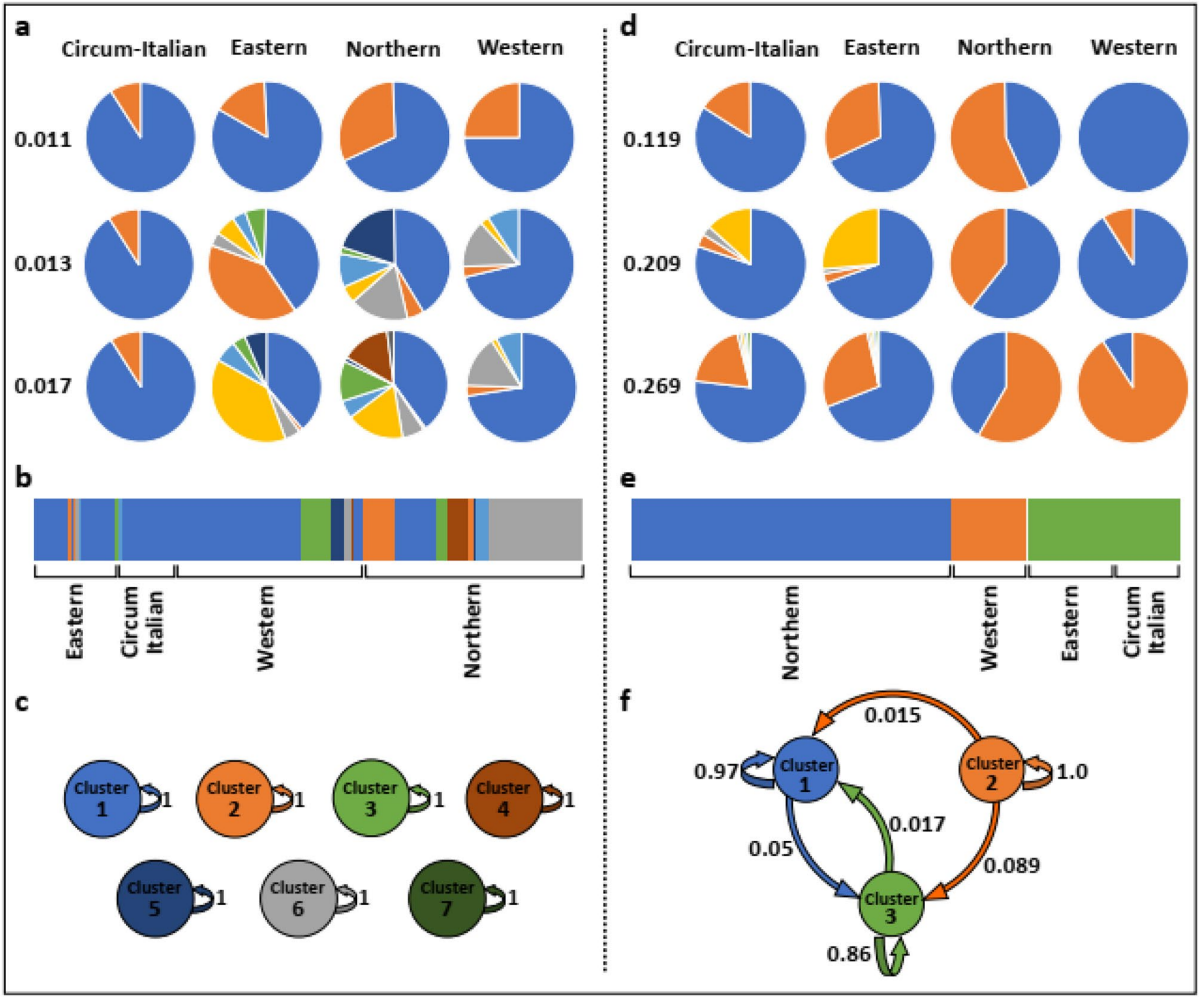
Population genetics analyses on *COI* haplotypes (**a–c**) and microsatellite alleles (**d–f**). (**a**) Netstruct results showing the number of clusters for three threshold values; (**b**) BAPS results**.** The seven clusters as distributed in the four maritime routes. Cluster identities are depicted in (**c**). Colour widths are proportional to the number of individuals within each cluster; (**c**) BAPS; zero geneflow rates among the clusters. (**d**) Netstruct results showing the number of clusters for three threshold values. (**e**) BAPS results**.** Clusters in each of the four maritime routes. The eastern and circum-Italian routes form one cluster. Colour widths are proportional to the number of individuals within the cluster. (**f)** Total number of clusters and geneflow rates among and within-clusters.

Fisher Exact test was then employed in two consecutive steps: the first, an overall test, resulted in highly significant differences (*p* = 0.000) and strong effect size (Cramer’s V = 0.57) among the routes. In the second, we analysed all six pairwise post-hoc tests between the four maritime routes, corrected for multiple testing, and revealed, again, high significant disparity of *COI* haplotypes between the routes (*p* = 0.000 Appendix Tables 3–9). The overall differentiation (PhiPT) value based on 999 permutations among the four maritime routes was 0.076 (*p* = 0.001) and pairwise PhiPT values ranged 0.054–0.120 (*p* < 0.002; Table 2). Based on Nei genetic distance the PcoA, with axes 1 and 2 capturing 93.7% of the explained variation, portrayed four distinct groups of haplotypes, fully matching the four maritime routes (Table 2; Fig. 2a). Next, a Netstruct analysis (Fig. 3a) was performed at ascending threshold levels between 0.001 to 0.017 (above which the network size of 818 samples collapsed) revealing two clusters of haplotypes for each maritime route at edge pruning threshold ranging 0.001–0.011, while at the highest edge pruning threshold of 0.017, 2,5,7, and 9 clusters appeared in the circum-Italian, the western, the eastern and the northern routes, respectively (*p* = 0.0001; Fig. 3a; Appendix Table 10), corresponding with haplotype numbers in the circum-Italian, and western routes (Table 1a), but not with the eastern and northern routes (9 and 11; Table 1a).

**Table 2:** *COI* and microsatellite analyses distinguishing the four major historical navigation routes in European waters: pairwise PhiPT/Fst, Nei genetic distances and Dest (for microsatellites alone). Probabilities, based on 999 permutations, are shown above diagonal.

	*COI* analyses				Microsatellites analyses			
	Pairwise population matrix of PhiPT values				Pairwise population matrix of Fst values			
	Eastern	Western	Northern	Circum-Italian	Eastern	Western	Northern	Circum-Italian
Eastern	–	0.001	0.001	0.004	–	0.001	0.001	0.001
Western	0.056	–	0.001	0.002	0.036	–	0.001	0.001
Northern	0.088	0.055	–	0.001	0.054	0.114	–	0.001
Circum-Italian	0.054	0.061	0.120	–	0.021	0.013	–	–

	Pairwise population matrix of Nei genetic distance values				Pairwise population matrix of Nei genetic distance Values			
	Eastern	Western	Northern	Circum-Italian	Eastern	Western	Northern	Circum-Italian
Eastern	–	–	–	–	–	–	–	–
Western	0.009	–	–	–	0.336	–	–	–
Northern	0.008	0.004	–	–	0.479	1.345	–	–
Circum-Italian	0.012	0.024	0.014	–	0.235	0.133	0.972	–

					Pairwise population matrix of Dest values			
					Eastern	Western	Northern	Circum-Italian
Eastern	–	–	–	–	–	0.001	0.001	0.001
Western	–	–	–	–	0.278	–	0.001	0.001
Northern	–	–	–	–	0.376	0.734	–	0.001
Circum–Italian	–	–	–	–	0.174	0.104	0.615	–

BAPS analysis (Fig. 3b) revealed 2, 5, 5 and 7 clusters in the circum-Italian, eastern, western and the northern routes, respectively, corresponding with haplotype numbers of the circum-Italian and western routes but not with the eastern and northern routes (Fig. 3b; Table 1a). The analysis further enabled us to calculate the amount of geneflow among the 7 emerged clusters, revealing the absence of any geneflow (Fig. 3c). Thus, although colonies that carry the same haplotypes or share identical cluster appear in more than one route, they probably do not admix. Netstruct and BAPS analyses further revealed that the number of clusters in the northern and eastern routes is smaller than the number of *COI* haplotypes (Table 1a).

### Microsatellites analyses

Using published results^21,23,25,29^, an inventory of 995 *Botryllus schlosseri* samples was analyzed on four microsatellite alleles: Pb29, Pb49, Pb41 and Bs811. These samples were collected from 32 sites, 5, 7, 20 in the eastern, western, and northern routes, respectively. Samples from 22 of these sites were also included in the *COI* analyses. In a similar way to the outcomes of *COI* haplotypes, the microsatellite alleles frequencies differed substantially among the routes (Appendix Table 15).

Fisher Exact test revealed that the four routes differed for each microsatellite locus (*p* = 0.000; Cramer’s V effect sizes range between moderate 0.33 to strong 0.60; Appendix Tables 11–14). Microsatellites basic information is summarized in Appendix Table 16. Overall population structure F_st_ value among the four maritime routes based on 999 permutations was 0.074 (*p* = 0.001) and pairwise F_st_ values varied between 0.013–0.114, (*p* = 0.001; Table 2). Overall Dest value was 0.396 (*p* << 0.05) and pairwise Dest ranged 0.104–0.734; *p* = 0.001 (Table 2). PcoA based on Nei genetic distance (Fig. 2b) portrayed four distinct groups of the microsatellite haplotypes, with axes 1 and 2 capturing 96.9% of the explained variation, fully matched with the assigned four maritime routes. Comparable to the *COI* outcomes, the northern route diverged by far from the others. Netstruct results showed 6 clusters at the threshold of 0.269 (Fig. 3d). BAPS results revealed 3 clusters (Fig. 3e), where two, are each restricted to northern and western routes and the third encompasses the eastern and circum-Italian together. Gene flow analyses (Fig. 3f) revealed that while there is a geneflow from cluster 2 (western route) to the other clusters, no geneflow goes to it. On the other hand, there is a geneflow between clusters 1 and 3 in both directions.

## Discussion

Human mediated dispersals are millennia-old phenomena^40^. These occurrences, not only facilitate erosions of the species biogeographic boundaries, but in addition directly alter seascape genetics of transported species^41,42^, despite the difficulty of disentangling between the impacts of natural processes and contributions by human-mediated dispersal mechanisms^24^. Associated with functional connectivity bearings, the balance between immigration of new genetic variants, and the extinction and diversification of existing genetic repertoire, are key factors that determine population genetics characteristics for decades to come^43^. Within this conceptualization, one of the leading drivers for the unintentional transfer of marine organisms is the global maritime traffic^44^, when long-term functional connectivity trajectories, may establish a ‘genetic memory’ of historical sea voyages and ancient population genetics characteristics. These seascape genetics may persist for centuries, leaving their footprints on present-day population genetics and furthermore serving as an important proxy for understanding the genetic assemblies of contemporary populations^45,46^. This is primarily true for species with limited ability for self-dispersal and limited larval mobility^47^ such as with *Botryllus schlosseri*^31^.

Using two independent molecular markers for genetic parameters (four nuclear microsatellite loci and 11 mitochondrial *COI* haplotypes) we analyzed here original and archive-mined data of 64 sampling sites (Appendix Table 1; the lack of *B. schlosseri* population genetic data from North African coasts is further noted), pertaining to coalesce into four European maritime routes (termed here as eastern, western northern and circum-Italian). We further performed identical population genetics tests (Fisher exact test, Fst and Dest, Nei genetic distance, Principal coordinate analysis, and two cluster analyses) on both genetic markers, with outcomes revealing the same results of significant segregation and differentiation between all routes, with the most conspicuous segregation for the northern route. The outcomes from the microsatellite analyses also uncovered an unexpected absence of gene flow toward the western route (Fig. 3f). This outcome could be partially clarified by the likelihood that abiotic factors, such as water temperatures and salinity, play a role in restructuring of *Botryllus schlosseri* seascape genetics, together with the influence of maritime traffic. As a support, Reem et al.^23^ identified genetic disparities between the eastern and western Mediterranean basins, as well as within the eastern basin of the Mediterranean Sea. These disparities were proposed to potentially arise from abiotic differences between the basins, which might slow down the rate of interconnectivity.

During Medieval times, maritime traffic in the Mediterranean was networked by the Genoa/Spain and Venice/Ottomans major trajectories^4,6,48^. The Genoa/Spain ships travelled mostly in the western and European-Atlantic routes, while the Venetian/Ottoman ships dominated the eastern route. While shipping routes in the Mediterranean Sea and European Atlantic commenced long ago, maritime transportation expanded dramatically as from the late 1500s^49^. Yet, the wind dependent ship velocity was slow (maximum 4–5 knots^14,50^ and did not change dramatically since ancient times^8,14^. The dependence on wind resulted in long journeys and frequent and long mooring breaks during periods of rough seas^50^. In contrast, the speed of modern vessels ranges between 20–26 knots, they are less sensitive to oceanographic conditions and are moored for much shorter periods than in the past due to advanced operation procedures in ports^51, 52^. Hundreds to thousands of slow-moving trade ships per year crossed the Mediterranean Sea and European Atlantic for centuries, to/from several major ports of origin (Appendix texts), while no evidence whatsoever has suggested that this traffic was halted in any period during the past ca. 3000 years. There is also no evidence showing that navigation routes have ever changed in historical times as a result of climatic changes^14^.

Maritime activities are associated with the transport of sedentary organisms that recruit to the ship hauls, a millennia-old phenomenon^38^, anecdotally documented in the literature for calcifying organisms e.g.,^15,53–58^. Yet, very little is known about the associations between the early sea voyages and the dispersal of fouling and boring communities in ancient wooden sea-going ships. Thus, the application of molecular biology methods, as performed here, may broaden our understanding on the impact of past invasions on current population genetics characteristics. In addition to the outcomes of natural phenomena, like post glacial impacts on genetics repertoires^25,59^, this study reveals that historical European intensive seafaring from the beginning of the Holocene, through the Bronze and Iron ages and toward the Classical Antiquity up to the industrial revolution have left their footprint on contemporary seascape genetics (revealed from the independent analyses of both, nuclear and mitochondrial markers) of one of the most common European urochordate, *Botryllus schlosseri*, a model case for presumable many other occurrences.

The study of seascape genetics aims to elucidate how spatially variable structural and environmental parameters (in the present study, intensive seafaring through major navigation routes) impact population genetic properties of marine organisms^60^. Past anthropogenic transport of species not only may impact genetic homogenization^61^, primarily under weak natural connectivity (such as with *Botryllus schlosseri*^21,30^), but may even result in constructed population genetics properties, ghost of past maritime trajectories, providing keys for understanding past, current and future seascape genetics.

### Supplementary Information

Supplementary Information.

## Data Availability

The dataset of microsatellites generated during the current study appear in Appendix Table 15, The accession numbers of the *COI* Haplotypes that are deposited in GenBank repository are found in appendix Table 2**.**
