# Supplementary material for: Historical navigation routes in European waters leave their footprint on the contemporary seascape genetics of a colonial urochordate
Source: Sci Rep. 2023 Nov 4;13:19076. doi: 10.1038/s41598-023-46174-0 (PMC10625628; doi:10.1038/s41598-023-46174-0)
Supplement: Supplementary file 1 — Supplementary Information. [file 41598_2023_46174_MOESM1_ESM.pdf]

Historical navigation routes in European waters leave their footprint on the contemporary seascape genetics of a colonial urochordate

Eitan Reem, Jacob Douek and Baruch Rinkevich

Appendix file

Content:

1. Appendix Tables 1-16.
2. Appendix texts: historical sources and references.

Appendix Tables 1-16

Appendix Table 1. Sampling sites of *B. schlosseri* populations used in the present study.
In bold: current study. In bold and Italics- sites where only microsatellites were analyzed. E=eastern route; W= western route; N= northern route; C=circum-Italian route.

No.	Location	Country	n	Region	Route	Collection source
1	Alicante	Spain	26	MED	W	[1], [2]
2	Arenys de Mar	Spain	19	MED	W	[1], [2]
3	Blanes	Spain	32	MED	W	[1],[2],[3]
4	Cadaques	Spain	25	MED	W	[3]
5	Cubelles	Spain	14	MED	W	[2],[3]
6	Estaque	France	32	MED	W	[1],[2],[3]
7	Estartit	Spain	17	MED	W	[2],[3],[4]
8	Naples	Italy	7	MED	W,C	[2]
9	**Glyfada**	**Greece**	**16**	**MED**	**E**	**[5], this study**
10	**Michmoret**	**Israel**	**73**	**MED**	**E**	**[5], this study**
11	**Ancona**	**Italy**	**31**	**MED**	**E,C**	**[5], this study**
12	**Carrara**	**Italy**	**52**	**MED**	**W,C**	**[5], this study**
13	**Palermo**	**Sicily**	**14**	**MED**	**W,E,C**	**[5], this study**
14	**Barcelona**	**Spain**	**14**	**MED**	**W**	**[5], this study**
15	**Gibraltar**	**Iberia(UK)**	**15**	**MED**	**W**	**[5], this study**
16	**Motril**	**Spain**	**8**	**MED**	**W**	**[5], this study**
17	**Rovinj**	**Croatia**	**30**	**MED**	**E**	**[5], this study**
18	**Marsaxlokk**	**Malta**	**15**	**MED**	**W**	**This study**
19	**Canet**	**France**	**9**	**MED**	**W**	[2],[4], **[5], this study**
20	Roses	Spain	11	MED	W	[3]
21	Sete	France	6	MED	W	[1], [2]
22	Venice	Italy	11	MED	E,C	[2], [4],[6]
23	Vilanova	Spain	3	MED	W	[3],[4],[7].
24	Ares	Spain	5	NEATL	N	[4]
25	**Brest**	**France**	**69**	**NEATL**	**N**	[2],[8], **this study**
26	Burela	Spain	8	NEATL	N	[4]
27	Cobh	Ireland	8	NEATL	N	[6]
28	Falmouth Harbor	England	58	NEATL	N	[2],[6]
29	Ferrol /Fornelos	Spain	15	NEATL	N	[4],[9]
30	Gijon	Spain	4	NEATL	N	[4]
31	Gosport	England	26	NEATL	N	[4]
32	Grana	Spain	7	NEATL	N	[1]
33	Granville	France	2	NEATL	N	[4]
34	Hamble Point	England	18	NEATL	N	[4]
35	La Rochelle	France	18	NEATL	N	[3],[4]
36	Llastres	Spain	6	NEATL	N	[4]
37	Mutriku	Spain	7	NEATL	N	[4]
38	Parkstone Bay	England	16	NEATL	N	[4]
39	Perros-Guirec	France	6	NEATL	N	[4]
40	**Plymouth**	**England**	**15**	**NEATL**	**N**	Ben Shlomo et al. 2006, Nydam et al. 2017, **this study**
41	Poole	England	14	NEATL	N	[4]
42	**Helgoland**	**Germany**	**18**	**NEATL**	**N**	[4],[8], **this study**
43	**Ålesund**	**Norway**	**18**	**NEATL**	**N**	**[10], This study**
44	**Florø**	**Norway**	**11**	**NEATL**	**N**	**[10], This study**
45	**Risor**	**Norway**	**11**	**NEATL**	**N**	**[10], This study**
46	**Ökerö island**	**Sweden**	**17**	**NEATL**	**N**	**[10], This study**
47	**Faro**	**Portugal**	**16**	**NEATL**	**N**	**This study**
48	**Sesimbra**	**Portugal**	**12**	**NEATL**	**N**	[8], **this study**
49	**Barbate**	**Spain**	**6**	**NEATL**	**N**	[8], **this study**
50	**Auchenmalg**	**England**	**10**	**NEATL**	**N**	[8], **this study**
51	**Lossiemouth**	**England**	**8**	**NEATL**	**N**	[8], **this study**
52	Port of Breskens	Holland	**7**	NEATL	N	[6]
53	**Roscoff**	**France**	**56**	**NEATL**	**N**	[2],[11], **this study**
54	Sada	Spain	4	NEATL	N	[4]
55	San Sebastian	Spain	4	NEATL	N	[4]

56	Santander	Spain	12	NEATL	N	[4]
57	Torquay	England	16	NEATL	N	[2],[4]
58	***Fiskebäckski***	***Sweden***	***25***	**NEATL**	*N*	***[10]***
59	***Hamburgsund***	***Sweden***	***18***	**NEATL**	*N*	***[10]***
60	***Strömstad***	***Sweden***	***48***	**NEATL**	*N*	***[10]***
61	***Fevik***	***Norway***	***30***	**NEATL**	***N***	***[10]***
62	***Tananger***	***Norway***	***18***	**NEATL**	***N***	***[10]***
63	***Håkonshell***	***Norway***	***40***	**NEATL**	***N***	***[10]***
64	***Dolvik***	***Norway***	***22***	**NEATL**	***N***	***[10]***

Literature cited for Appendix Table 1

1.Lejeusne, C., Bock D.G., Therriault, T.W., MacIsaac, H.J. & Cristescu, M.E. Comparative phylogeography of two colonial ascidians reveals contrasting invasion histories in North America, *Biol Invasions* **13**, 635-650 (2011).

2.Bock, D.G., MacIsaac, H.J. & Cristescu, M.E. Multilocus genetic analyses differentiate between widespread and spatially restricted cryptic species in a model ascidian, *Proc R Soc B.* **279**, 2377-238 (2012).

3.López-Legentil, S., Turon, X. & Planes, S. Genetic structure of the star sea squirt, *Botryllus schlosseri*, introduced in southern European harbours, *Mol Ecol.* ***15***, 3957-3967 (2006).

4.Nydam, M.L., Giesbrecht, K.B. & Stephenson E.E. Origin and dispersal history of two colonial ascidian clades in the *Botryllus schlosseri* species complex. *Plos One* **12**, e0169944 (2017).

5.Reem, E., Douek, J., Paz, G., Katzir, G. & Rinkevich B. Phylogenetics Biogeography and Population genetics, of the ascidian *Botryllus schlosseri* in the Mediterranean Sea and beyond. *Mol Phylogenet Evol*. **107**, 221-231 (2017).

6.Yund, P.O., Collins, C. & Johnson, S.L. Evidence of a Native Northwest Atlantic COI Haplotype Clade in the Cryptogenic Colonial Ascidian *Botryllus schlosseri. Biol Bull.* **228**, 201-216 (2015).

7.López-Legentil, S., Legentil, M.L., Erwin, P.M & Turon, X. Harbor networks as introduction gateways: contrasting distribution patterns of native and introduced ascidians. *Biol. Invasions*. **17**, 1623-1638 (2015).

8.Ben-Shlomo, R., Paz, G. & Rinkevich, B. Postglacial-period and recent invasions shape the population genetics of botryllid ascidians along European Atlantic coasts. *Ecosystems* **9**, 1118-1127 (2006).

9.Pérez-Portela, R., Bishop, J.D.D., Davis, A.R. and Turon, X. Phylogeny of the families Pyuridae and Styelidae (Stolidobranchiata, Ascidiacea) inferred from mitochondrial and nuclear DNA sequences. *Mol Phylogenet Evol*. **50**, 560-575 (2009).

10.Reem, E., Mohanty, I., Katzir, G. & Rinkevich, B. Population genetic structure and modes of dispersal for the colonial ascidian *Botryllus schlosseri* along the Scandinavian Atlantic coasts. *Marine Ecology Progress Series,* **485**, 143–154 (2013).

11.Stach, T., Turbeville, J.M. Phylogeny of Tunicata inferred from molecular and morphological characters. *Mol Phylogenet Evol.* **25**, 408-428 (2002).

Appendix Table 2. COI haplotypes used for the study with accession numbers.

COI haplotype	Accession no.
4	DQ340205
5	DQ340211
6	DQ340209
7	DQ223768
9	MN064602
22	KM587652
24	MN718180
25	KY235421
30	KX500818
67	GQ365703
71	DQ340216

Appendix Table 3. Fisher Exact using COI haplotypes test results for the four navigation routes. 1= eastern, 2=circum Italian, 3= western, 4= northern. The same holds for all the Tables 3-9.

	Value	df	Asymp. Sig. (2-sided)	Monte Carlo Sig. (2-sided)		
				Sig.	99% Confidence Interval	
					Lower Bound	Upper Bound
**Pearson Chi-Square**	799.020[a]	30	0.000	0.000[b]	0.000	0.000
**Likelihood Ratio**	778.516	30	0.000	0.000[b]	0.000	0.000
**Fisher's Exact Test**	723.650	-	-	0.000[b]	0.000	0.000
**No of Valid Cases**	818	-	-	-	-	-
**Cramer's V Effect size**	0.571	-	-	-	-	-

a. 17 cells (38.6%) have expected count less than 5. The minimum expected count is .52.

b. Based on 100000 sampled tables with starting seed 403768731.

Appendix Table 4. Route 1 vs. Route 2 significance results.

	Value	df	Asymp. Sig. (2-sided)	Monte Carlo Sig. (2-sided)		
				Sig.	99% Confidence Interval	
					Lower Bound	Upper Bound
**Pearson Chi-Square**	72.141[a]	8	0.000	0.000[b]	0.000	0.000
**Likelihood Ratio**	96.587	8	0.000	0.000[b]	0.000	0.000
**Fisher's Exact Test**	84.008	-	-	0.000[b]	0.000	0.000
**No of Valid Cases**	210	-	-	-	-	-
**Cramer's V Effect size**	0.586	-	-	-	-	-

a. 12 cells (66.7%) have expected count less than 5. The minimum expected count is .40.

b. Based on 100000 sampled tables with starting seed 1487459085.

Appendix Table 5. Route 1 vs. route 3 significance results.

	Value	df	Asymp. Sig. (2-sided)	Monte Carlo Sig. (2-sided)		
				Sig.	99% Confidence Interval	
					Lower Bound	Upper Bound
**Pearson Chi-Square**	181.312[a]	10	0.000	0.000[b]	0.000	0.000
**Likelihood Ratio**	199.207	10	0.000	0.000[b]	0.000	0.000
**Fisher's Exact Test**	183.482	-	-	0.000[b]	0.000	0.000
**No of Valid Cases**	403	-	-	-	-	-
**Cramer's V Effect size**	0.671	-	-	-	-	-

a. 13 cells (59.1%) have expected count less than 5. The minimum expected count is .31.

b. Based on 100000 sampled tables with starting seed 1421288173.

Appendix Table 6. Route 1 vs. route 4 significance results.

	Value	df	Asymp. Sig. (2-sided)	Monte Carlo Sig. (2-sided)		
					99% Confidence Interval	
				Sig.	Lower Bound	Upper Bound
**Pearson Chi-Square**	225.714[a]	10	0.000	0.000[b]	0.000	0.000
**Likelihood Ratio**	248.025	10	0.000	0.000[b]	0.000	0.000
**Fisher's Exact Test**	237.148	-	-	0.000[b]	0.000	0.000
**No of Valid Cases**	455	-	-	-	-	-
**Cramer's V Effect size**	0.704	-	-	-	-	-

a. 6 cells (27.3%) have expected count less than 5. The minimum expected count is 1.10

b. Based on 100000 sampled tables with starting seed 1122541128.

Appendix Table 7. Route 2 vs. route 3 significance results.

	Value	df	Asymp. Sig. (2-sided)	Monte Carlo Sig. (2-sided)		
					99% Confidence Interval	
				Sig.	Lower Bound	Upper Bound
**Pearson Chi-Square**	49.645[a]	5	0.000	0.000[b]	0.000	0.000
**Likelihood Ratio**	64.487	5	0.000	0.000[b]	0.000	0.000
**Fisher's Exact Test**	51.986	-	-	0.000[b]	0.000	0.000
**No of Valid Cases**	363	-	-	-	-	-
**Cramer's V Effect size**	0.370	-	-	-	-	-

a. 4 cells (33.3%) have expected count less than 5. The minimum expected count is .70.

b. based on 100000 sampled tables with starting seed 1201206483.

Appendix Table 8. Route 2 vs. route 4 significance results

	Value	df	Asymp. Sig. (2-sided)	Monte Carlo Sig. (2-sided)		
					99% Confidence Interval	
				Sig.	Lower Bound	Upper Bound
**Pearson Chi-Square**	201.611[a]	10	0.000	0.000[b]	0.000	0.000
**Likelihood Ratio**	223.710	10	0.000	0.000[b]	0.000	0.000
**Fisher's Exact Test**	202.729	-	-	0.000[b]	0.000	0.000
**No of Valid Cases**	415	-	-	-	-	-
**Cramer's V Effect size**	0.697	-	-	-	-	-

a. 10 cells (45.5%) have expected count less than 5. The minimum expected count is .20.

b. Based on 100000 sampled tables with starting seed 745618922.

Appendix Table 9. Route 3 vs. route 4 significance results.

	Value	df	Asymp. Sig. (2-sided)	Monte Carlo Sig. (2-sided)		
					99% Confidence Interval	
				Sig.	Lower Bound	Upper Bound
**Pearson Chi-Square**	343.492[a]	10	0.000	0.000[b]	0.000	0.000
**Likelihood Ratio**	412.354	10	0.000	0.000[b]	0.000	0.000
**Fisher's Exact Test**	394.249	-	-	0.000[b]	0.000	0.000
**No of Valid Cases**	608	-	-	-	-	-
**Cramer's V Effect size**	0.752	-	-	-	-	-

a. 7 cells (31.8%) have expected count less than 5. The minimum expected count is .46.
b. Based on 100000 sampled tables with starting seed 205597102.

Appendix Table 10. NetStruct analysis on COI haplotypes and clusters for each maritime route, including different edge pruning levels.

Threshold/edge pruning level	Eastern route: 9 haplotypes	Circum Italy route: 2 haplotypes	Western route: 5 haplotypes	Norththern route: 11 haplotypes
0.011	2	2	2	2
0.013	6	2	5	7
0.017	7	2	5	9

Appendix Table 11. Pb29 microsatellite locus effect size and significance results on SPSS.

	Value	df	Asymptotic Significance (2-sided)	Monte Carlo Sig. (2-sided)		
					99% Confidence Interval	
				Significance	Lower Bound	Upper Bound
Pearson Chi-Square	338.074[a]	54	0.000	0.000[b]	0.000	0.000
Likelihood Ratio	393.840	54	0.000	0.000[b]	0.000	0.000
Fisher's Exact Test	380.226	-	-	0.000[b]	0.000	0.000
Linear-by-Linear Association	71.685[c]	1	0.000	0.000[b]	0.000	0.000
No of Valid Cases	1017	-	-	-	-	-
Cramer’s V effect size	0.333	-	-	-	-	-

55 cells (72.4%) have expected count less than 5. The minimum expected count is .14.[a]

Based on 10000 sampled tables with starting seed 2000000.[b]

The standardized statistic is -8.467.[c]

Appendix Table 12. Pb49 microsatellite locus effect size and significance results on SPSS

	Value	df	Asymptotic Significance (2-sided)	Monte Carlo Sig. (2-sided)		
					99% Confidence Interval	
				Significance	Lower Bound	Upper Bound
Pearson Chi-Square	879.044[a]	144	0.000	0.000[b]	0.000	0.000
Likelihood Ratio	996.053	144	0.000	0.000[b]	0.000	0.000
Fisher's Exact Test	911.398	-	-	0.000[b]	0.000	0.000
Linear-by-Linear Association	287.689[c]	1	0.000	0.000[b]	0.000	0.000
No of Valid Cases	898	-	-	-	-	-
Cramer’s V effect size	0.571	-	-	-	-	-

152 cells (77.6%) have expected count less than 5. The minimum expected count is .13.[a]

Based on 10000 sampled tables with starting seed 743671174.[b]

The standardized statistic is 16.961.[c]

Appendix Table 13. Pb41 microsatellite locus effect size and significance results on SPSS

	Value	df	Asymptotic Significance (2-sided)	Monte Carlo Sig. (2-sided)		
					99% Confidence Interval	
				Significance	Lower Bound	Upper Bound
**Pearson Chi-Square**	614.309[a]	105	0.000	0.000[b]	0.000	0.000
**Likelihood Ratio**	685.745	105	0.000	0.000[b]	0.000	0.000
**Fisher's Exact Test**	628.281	-	-	0.000[b]	0.000	0.000
**Linear-by-Linear Association**	151.041[c]	1	0.000	0.000[b]	0.000	0.000
**No of Valid Cases**	927	-	-	-	-	-
**Cramer's V effect size**	0.470	-	-	-	-	-

105 cells (72.9%) have expected count less than 5. The minimum expected count is .13.[a]

Based on 10000 sampled tables with starting seed 329836257.[b]

The standardized statistic is 12.290.[c]

Appendix Table 14. Bs811 microsatellite locus effect size and significance results on SPSS

	Value	df	Asymptotic Significance (2-sided)	Monte Carlo Sig. (2-sided)		
					99% Confidence Interval	
				Significance	Lower Bound	Upper Bound
**Pearson Chi-Square**	966.163[a]	294	0.000	0.000[b]	0.000	0.000
**Likelihood Ratio**	1012.845	294	0.000	0.000[b]	0.000	0.000
**Fisher's Exact Test**	881.770	-	-	0.000[b]	0.000	0.000
**Linear-by-Linear Association**	42.293[c]	1	0.000	0.000[b]	0.000	0.000
**No of Valid Cases**	888	-	-	-	-	-
**Cramer's V effect size**	0.602	-	-	-	-	-

351 cells (88.6%) have expected count less than 5. The minimum expected count is .13.[a]

Based on 10000 sampled tables with starting seed 215962969.[b]

The standardized statistic is 6.503.[c]

Appendix Table 15

Microsatellite haplotypes Frequencies and Sample Size by route.

Locus	Allele/n	Northern	Western	Eastern	Circum Italian
**PB29**	**N**	**564**	**140**	**154**	**120**
	**144**	0.000	0.004	0.000	0.000
	**150**	0.000	0.004	0.000	0.004
	**151**	0.003	0.025	0.006	0.013
	**152**	0.164	0.032	0.114	0.163
	**153**	0.092	0.496	0.347	0.333
	**154**	0.002	0.000	0.000	0.000
	**155**	0.217	0.000	0.143	0.150
	**156**	0.152	0.414	0.179	0.292
	**157**	0.003	0.000	0.000	0.000
	**158**	0.007	0.000	0.003	0.004
	**159**	0.002	0.004	0.006	0.008
	**162**	0.343	0.007	0.188	0.029
	**163**	0.003	0.004	0.000	0.004
	**164**	0.003	0.000	0.000	0.000
	**165**	0.003	0.000	0.000	0.000
	**166**	0.003	0.011	0.000	0.000
	**169**	0.000	0.000	0.013	0.000
	**170**	0.003	0.000	0.000	0.000
	**189**	0.002	0.000	0.000	0.000
**PB49**	**N**	**527**	**121**	**135**	**111**
	**176**	0.182	0.000	0.000	0.000
	**190**	0.001	0.000	0.000	0.000
	**195**	0.031	0.000	0.000	0.000
	**197**	0.004	0.000	0.000	0.000
	**198**	0.001	0.000	0.000	0.000
	**199**	0.002	0.000	0.000	0.000
	**200**	0.001	0.000	0.007	0.009
	**201**	0.109	0.000	0.011	0.000
	**203**	0.008	0.000	0.004	0.000
	**204**	0.000	0.000	0.007	0.009
	**205**	0.142	0.017	0.033	0.000
	**207**	0.011	0.000	0.004	0.000
	**208**	0.000	0.004	0.000	0.000
	**209**	0.139	0.000	0.011	0.014
	**210**	0.000	0.012	0.000	0.009
	**211**	0.190	0.029	0.048	0.059
	**212**	0.000	0.112	0.122	0.050
	**213**	0.082	0.008	0.070	0.018
	**214**	0.000	0.017	0.037	0.032
	**215**	0.005	0.000	0.004	0.005
	**216**	0.000	0.008	0.000	0.000

	218	0.000	0.008	0.007	0.000
	219	0.005	0.000	0.000	0.000
	220	0.000	0.025	0.037	0.023
	221	0.029	0.000	0.000	0.000
	222	0.000	0.050	0.126	0.050
	223	0.003	0.000	0.000	0.000
	224	0.000	0.087	0.070	0.126
	225	0.009	0.000	0.000	0.000
	226	0.000	0.099	0.026	0.045
	227	0.004	0.000	0.000	0.000
	228	0.003	0.033	0.044	0.059
	229	0.008	0.008	0.004	0.005
	230	0.000	0.045	0.052	0.068
	231	0.003	0.066	0.015	0.059
	232	0.000	0.045	0.033	0.090
	233	0.004	0.045	0.000	0.009
	234	0.000	0.008	0.067	0.009
	235	0.019	0.050	0.015	0.009
	236	0.000	0.004	0.056	0.050
	237	0.005	0.041	0.004	0.032
	238	0.000	0.004	0.000	0.005
	239	0.002	0.083	0.048	0.081
	241	0.000	0.045	0.022	0.045
	242	0.000	0.008	0.000	0.000
	243	0.000	0.012	0.015	0.018
	245	0.000	0.012	0.000	0.009
	246	0.000	0.008	0.000	0.005
	248	0.000	0.004	0.000	0.005
**PB41**	**N**	**534**	**140**	**152**	**118**
	156	0.103	0.000	0.000	0.000
	161	0.002	0.007	0.000	0.008
	162	0.017	0.000	0.000	0.000
	163	0.002	0.000	0.000	0.000
	164	0.022	0.000	0.000	0.000
	165	0.010	0.000	0.000	0.000
	166	0.012	0.000	0.000	0.000
	167	0.037	0.004	0.016	0.025
	168	0.008	0.000	0.000	0.000
	169	0.408	0.000	0.197	0.034
	170	0.030	0.043	0.007	0.008
	171	0.049	0.075	0.079	0.174
	172	0.070	0.207	0.118	0.123
	173	0.000	0.061	0.007	0.017
	174	0.112	0.046	0.161	0.072
	175	0.056	0.071	0.026	0.034
	176	0.007	0.071	0.056	0.076
	177	0.015	0.032	0.092	0.030
	178	0.005	0.179	0.092	0.267
	179	0.004	0.132	0.095	0.068
	180	0.004	0.014	0.007	0.025
	181	0.000	0.025	0.000	0.000

	182	0.003	0.004	0.000	0.000
	183	0.009	0.000	0.000	0.000
	184	0.011	0.000	0.007	0.008
	186	0.000	0.000	0.033	0.008
	187	0.000	0.004	0.000	0.004
	188	0.000	0.004	0.000	0.000
	190	0.002	0.004	0.000	0.000
	191	0.000	0.004	0.000	0.004
	196	0.000	0.004	0.000	0.004
	198	0.000	0.004	0.000	0.000
	199	0.002	0.000	0.000	0.000
	209	0.000	0.007	0.000	0.000
	210	0.000	0.000	0.003	0.004
	212	0.000	0.000	0.003	0.004
**Bs811**	**N**	**501**	**131**	**141**	**116**
	162	0.001	0.000	0.000	0.000
	164	0.008	0.000	0.000	0.000
	166	0.005	0.000	0.000	0.000
	169	0.028	0.000	0.000	0.000
	171	0.000	0.004	0.007	0.000
	172	0.026	0.000	0.000	0.000
	173	0.000	0.019	0.007	0.009
	174	0.197	0.023	0.000	0.000
	175	0.002	0.008	0.007	0.000
	176	0.039	0.015	0.018	0.009
	177	0.000	0.000	0.035	0.026
	178	0.122	0.019	0.043	0.017
	179	0.000	0.000	0.035	0.043
	180	0.009	0.000	0.000	0.000
	181	0.000	0.000	0.021	0.009
	182	0.000	0.000	0.021	0.026
	184	0.023	0.011	0.000	0.000
	185	0.000	0.000	0.004	0.004
	186	0.083	0.008	0.011	0.013
	187	0.000	0.004	0.014	0.017
	188	0.001	0.000	0.011	0.013
	189	0.002	0.000	0.007	0.009
	190	0.017	0.000	0.018	0.009
	191	0.000	0.008	0.000	0.000
	192	0.004	0.008	0.000	0.000
	193	0.000	0.000	0.018	0.022
	195	0.006	0.000	0.021	0.026
	196	0.022	0.000	0.014	0.000
	197	0.012	0.000	0.050	0.039
	198	0.003	0.000	0.000	0.000
	199	0.002	0.000	0.021	0.009
	200	0.029	0.000	0.000	0.000
	201	0.000	0.000	0.021	0.026
	202	0.063	0.000	0.014	0.000
	203	0.002	0.019	0.028	0.034
	204	0.004	0.011	0.000	0.004

**205**	0.000	0.000	0.053	0.022
**206**	0.032	0.008	0.014	0.017
**207**	0.002	0.004	0.071	0.000
**208**	0.007	0.000	0.000	0.000
**209**	0.009	0.023	0.021	0.009
**210**	0.008	0.004	0.021	0.009
**211**	0.008	0.095	0.011	0.022
**212**	0.006	0.000	0.032	0.000
**213**	0.002	0.023	0.004	0.030
**214**	0.024	0.008	0.064	0.000
**215**	0.000	0.172	0.025	0.147
**216**	0.011	0.000	0.046	0.017
**217**	0.000	0.145	0.018	0.108
**218**	0.018	0.004	0.000	0.000
**219**	0.035	0.050	0.000	0.039
**220**	0.009	0.000	0.000	0.000
**221**	0.002	0.015	0.004	0.022
**223**	0.000	0.008	0.000	0.009
**224**	0.000	0.000	0.004	0.004
**225**	0.000	0.015	0.025	0.017
**227**	0.002	0.031	0.014	0.030
**229**	0.002	0.004	0.007	0.009
**230**	0.008	0.000	0.007	0.000
**231**	0.000	0.053	0.018	0.034
**232**	0.000	0.000	0.004	0.004
**233**	0.000	0.031	0.007	0.004
**235**	0.003	0.080	0.000	0.034
**236**	0.008	0.000	0.000	0.000
**237**	0.001	0.027	0.007	0.022
**238**	0.005	0.000	0.000	0.000
**239**	0.002	0.023	0.000	0.017
**240**	0.004	0.000	0.000	0.000
**241**	0.000	0.011	0.000	0.009
**242**	0.002	0.000	0.000	0.000
**243**	0.004	0.000	0.004	0.000
**245**	0.000	0.000	0.007	0.000
**247**	0.009	0.000	0.000	0.000
**248**	0.002	0.000	0.000	0.000
**249**	0.015	0.000	0.000	0.000
**250**	0.003	0.000	0.000	0.000
**251**	0.006	0.000	0.000	0.000
**252**	0.001	0.000	0.000	0.000
**253**	0.004	0.000	0.000	0.000
**254**	0.007	0.000	0.000	0.000
**255**	0.003	0.000	0.000	0.000
**257**	0.000	0.004	0.000	0.004
**258**	0.001	0.000	0.000	0.000
**259**	0.000	0.000	0.035	0.000
**261**	0.002	0.008	0.000	0.000
**265**	0.001	0.000	0.000	0.000
**268**	0.003	0.000	0.000	0.000

**271**	0.000	0.000	0.007	0.000
**272**	0.002	0.000	0.000	0.000
**273**	0.000	0.000	0.014	0.000
**274**	0.002	0.000	0.004	0.000
**278**	0.001	0.000	0.000	0.000
**280**	0.001	0.000	0.000	0.000
**286**	0.002	0.000	0.000	0.000
**288**	0.000	0.000	0.007	0.000
**303**	0.002	0.000	0.000	0.000
**305**	0.008	0.000	0.000	0.000
**306**	0.002	0.000	0.000	0.000
**307**	0.001	0.000	0.000	0.000

Appendix Table 16

Microsatellite basic information. **Na** number of alleles, **Ho** observed heterozygosity, **He** expected heterozygosity, **AR** allelic richness, **F** fixation index.

a. basic information per route

Pop	N	Na	Ho	He	AR	F
**Northern**	531.50	35.00	0.24	0.84	24.20	0.71
**Western**	133.00	25.00	0.48	0.83	23.93	0.37
**Eastern**	145.50	26.75	0.37	0.89	25.68	0.58
**Circum**	116.25	26.25	0.47	0.88	26.05	0.45
**Mean**	231.56	28.25	0.39	0.86	33.02	0.53

b. basic information per locus in each route

Pop	Locus	N	Na	Ho	He	AR	F
**northern**	**PB29**	564	16.00	0.28	0.78	10.27	0.64
	**PB49**	527	27.00	0.33	0.87	19.35	0.62
	**PB41**	534	25.00	0.22	0.80	20.26	0.73
	**Bs811**	501	72.00	0.15	0.93	46.93	0.84
**western**	**PB29**	140	10.00	0.64	0.58	9.12	-0.11
	**PB49**	121	31.00	0.41	0.94	30.62	0.56
	**PB41**	140	22.00	0.65	0.88	20.26	0.26
	**Bs811**	131	37.00	0.21	0.92	35.74	0.78
**eastern**	**PB29**	154	9.00	0.53	0.78	8.56	0.32
	**PB49**	135	29.00	0.34	0.94	28.01	0.64
	**PB41**	152	17.00	0.40	0.88	16.17	0.55
	**Bs811**	141	52.00	0.20	0.97	49.98	0.79
**circum**	**PB29**	120	10.00	0.64	0.75	9.77	0.15
	**PB49**	111	30.00	0.43	0.94	30.00	0.54
	**PB41**	118	21.00	0.58	0.86	20.69	0.33
	**Bs811**	116	44.00	0.22	0.95	43.72	0.76

Appendix Texts

Appendix Text 1: Selected literature sources and citations on historic maritime routes. Only main citations and major summaries/conclusions pertaining the manuscript topic are provided.

1. Balard M, (2016) Coastal Shipping and Navigation in the Mediterranean. In: Stuckey J, (Ed.), The Eastern Mediterranean Frontier of Latin Christendom Routledge Taylor &Francis Group London & New York.

"Ever since Phoenician times, the Mediterranean has been a hospitable sea, allowing easy contact between the cities of the Greek world dotted around its shores. The steep slopes on its northern coasts combined with the difficulties of transportation by land, notwithstanding the extensive network of Roman roads, made the Mediterranean the most effective link between these coastal cities. The Middle Ages inherited innumerable maritime traditions from Greek and Roman times. From the tenth century onwards, Italian cities found themselves in the vanguard of a revival in seafaring. This is how Italy came to be one of the most powerful countries in the region. Italy seemed to be the source of everything: not only was she responsible for the major inventions, but she then spread the use of these inventions, firstly in the direction of the western basin of the Mediterranean, and later, from the thirteenth century onwards, towards the Atlantic coasts of Spain and Portugal".

"During the Middle Ages, navigation in the Mediterranean was still subject to certain limitations. It was a region where landmarks such as promontories and islands, and the enforced use of certain channels, made navigation easy, and sailors were reluctant to let the coast slip from view. Mediterranean ships rarely ventured far from the coast. In fact, their voyages largely consisted of moving from one natural landmark to the next. For the most part they relied on points on the coastline that seafarers had come to know from tradition and experience, and which had been noted down in portulans and charts. Navigation in the Mediterranean was mainly of the coastal variety, and was also fundamentally empirical."

"In the fifteenth century, no more than about ten Venetian galleys arrived each year in Alexandria, and only three or four Genoese or Catalonian ships; other maritime states of the western Mediterranean such as Ancona, Ragusa, Marseilles and Montpellier sent ships only very irregularly."

"Small and medium-sized vessels played an essential role in this network throughout the east and the west of the Mediterranean. In fact, coastal fleets were particularly active in Genoa's overseas colonies."

"After 1277, the Genoese were the first to establish regular links between the Mediterranean and Flanders passing through the Straits of Gibraltar."

"In the Adriatic: Between 1596 and 1600 as many as 68 sailing ships were counted in the Bay of Levkimmi on Corfu. As many as 398 vessels were identified between 1563 and 1591 sailing in all directions."

2. Pryor J.H. (1995). The Geographical conditions of Galley navigation in the Mediterranean. In: The Age of the Galley: Mediterranean Oared Vessels Since Pre-Classical Times (Conway's History of the Ship) by Robert Gardiner (Ed.). Conway Maritime Press, London.

"The historical record provides no evidence whatsoever to suggest that in any period during the past 2500 years climatic variation causes changes in weather patterns which would invalidate any conclusions drawn from modern observations about the influence of meteorological conditions on navigation. Ancient and medieval observations of wind directions invariably coincide with the evidence of modern meteorological data. The same is true of the current circulation in the Mediterranean and the tides experienced in certain narrows and bights. There is no evidence that the conditions of navigation have ever changed significantly in historical times as a result of climatic changes."

3: Jacoby D, (2016) Byzantine Crete in the Navigation and Trade Networks of Venice and Genoa. In: Stuckey J, (Ed.), The Eastern Mediterranean Frontier of Latin Christendom Routledge Taylor &Francis Group London & New York.

This source reveals the importance of Venice in the eastern trade routes: "By the ninth century Venice was already conducting fairly regular trade with both Byzantium or Romania and the Islamic Levant."

4. Kapetanakis PS, (2010). Gauging Maritime Trade between the Mediterranean and Northern Europe in the Late Eighteenth and Mid-Nineteenth Centuries, Using Electronic Databases Journal of Mediterranean Studies, Volume 19, Number 2, pp. 295-310

"During the second half of the eighteenth century, the Mediterranean had started experiencing a come-back in the Western European economy and by the mid-nineteenth century the Mediterranean had become one of the busiest and most important commercial arteries of Europe and the world" (page 298).

While before the eighteen century the number of commercial vessels that sailed between the Mediterranean and the Baltic Sea was limited to about 50/year (see section 6 below), **"**the number of merchant vessels departing from the Baltic Sea for Mediterranean ports shows an upward trend, at least as regards the earlier part of our period with the number of vessels increasing from 157 in 1784, to 243 vessels in 1787, falling to an average of around 200 vessels per annum for the years 1788–1791, before climbing to 234 vessels in 1792" (page 300). During the period 1784 to 1795, 2057 (171/year) vessels arrived to the Baltic from the Twenty most important Mediterranean Trading Ports for the Baltic (page 301). This reference further reveals that maritime trajectories between the Baltic and the Mediterranean were almost exclusively with the Spanish, French and Italian ports of the Western Mediterranean (pages 301-302).

5. Munroe JH, (1999). The Low Countries' Export Trade in Textiles with the Mediterranean Basin, 1200-1600: A Cost-Benefit Analysis of Comparative Advantages in Overland and Maritime Trade Routes. International Journal of Maritime History, Vol. XI, No. 2 1-30

"As early as c. 1274, the Genoese had made the first direct maritime contact with Flanders; and by 1317 the Italians had instituted a fairly regularannual galley service to Bruges. The Venetians, after founding their Bruges consulate in 1322 and establishing a maritime link with Southampton, came to dominate that northern galley trade."

6. Pourchasse P (2011) Trade between France and Sweden in the Eighteenth Century. Forum navale 67, 92-114.

Data is given for foreign ships arriving to Marseilles along 60 years (1730-1790 BC)

Dates	Dutch	English	Danish and Swedish
1730–1739	244	748	37
1740–1749	685	298	195
1750–1759	606	588	390
1760–1769	786	331	424
1770–1179	682	482	513
1780–1789	261	260	1106
**Total**	**3264**	**2707**	**2665**
**Mean/year**	**55**	**46**	**45**

Data is provided for transportation of French colonial products, primarily from Marseilles to the Baltic 1784–1789

	**Sugar (Tons)**	**Coffee (Tons)**	**Indigo (Kg)**
Copenhagen	737.7	3,217.5	23,621.3
Lübeck	160.4	178.1	2,743.6
Stralsund	85.6	303.0	1,482.0
Stettin	17,879.8	2,923.4	29,191.3
Danzig	3,722.0	2,342.1	13,220.3
Königsberg	1,298.0	622.8	51,341.7
Riga	629.1	373.2	1,788.1
St Petersburg	9,330.1	1,115.2	40,312.9
Stockholm	1,532,5	577.6	14,058.6
**Total**	**32,025.4**	**11,652.9**	**177,759.8**
Estimated number of ships that loaded the products*	40	15	<1

*Based on 800 tons/ship, 55 ships carried the above merchandise during five years and 11/year.

7. Rickman GE (1980). The Grain Trade under the Roman Empire. Memoirs of the American Academy in Rome, Vol. 36, The Seaborne Commerce of Ancient Rome: Studies in Archaeology and History, pp. 261-275.

Summary: During the period of the Roman Empire (27 BC- 476 AD) the capital populations consumed large quantities of wheat and barley. It is estimated that Rome needed more than 250000 tons of grains/year. The supply of grains from Egypt was about 87000 tons/year. The capacity of ships that carried the grains ranged between 340-1200 tons/ship. Only few ships reached the capacity of 1200 tons and most of the ships were small. Assuming an average capacity of 600 tons/ship we calculated the traffic of 140-150 ships/year that carried grains from Alexandria to Rome.

8. Van Gelder M (2009) Trading Places: The Netherlandish Merchants in Early Modern Venice. Library of economic history. Brill publishing house.

Chapter 2, page 59 footnote 45: “Van Royen, “The first phase” page 87. Van Royen arrives at a total of 115 freight contracts made in Amsterdam for Italy between 1591 and 1594. The years with a high number of charter-parties coincide with periods of grain shortages in Italy (1592–1594, 1606–1607). Cf. Engels, *Merchants, interlopers*, pages 83–88. For Livorno and Genoa registers of the customs offices and *Sanità*, the office listing all ships coming from areas with high risks of contagious diseases, exist, which can give an insight into the number of incoming ships. Between 1590 and 1593 a total of 227 ships from northern Europe arrived in Livorno, Braudel and Romano, *Navires et marchandises*, page 51. During the period 1590–1594, it seems that some four hundred ships from that same region sailed to Genoa, Grendi, “I Nordici”, page 35. The numbers of ships reaching Genoa and Livorno cannot be simply added up, as ships frequently tended to call in both ports in the course of the same journey”.

“Between 1591 and 1609 forty-six immigrant traders in Amsterdam were involved in grain exports to Italy, freighting 569 ships and taking care of sixty-fi ve per cent of the total number of shipments. In 1606–1607, years in which Italy again was suff ering from extreme food shortages, the four Antwerp merchants Casper

Quingetti, Jacques de Velaer, Isaac Lemaire, and Jan Calandrini together sent 250 ships to the Mediterranean”.

“A total of 280,000 *staio* of wheat and 40,000 *staio* of rye was imported in 1607”. 1staio = 25.4 kg. That means 320000 staio =8128 tons. In order to carry this amount more than 20 ships were needed.

Chapter 3: “Many shipmasters from the Netherlands criss-crossed the Mediterranean looking for profitable cargo. Claes Pietersen of the ‘Hercules’, for instance, sailed a couple of times between Venice and Cyprus before returning to Amsterdam in August 1606”. (Page 75)

" Ninety ships sailed from Amsterdam to the Mediterranean, while only thirty-nine arrivals have been recorded”. (Page 79 footnote 52)

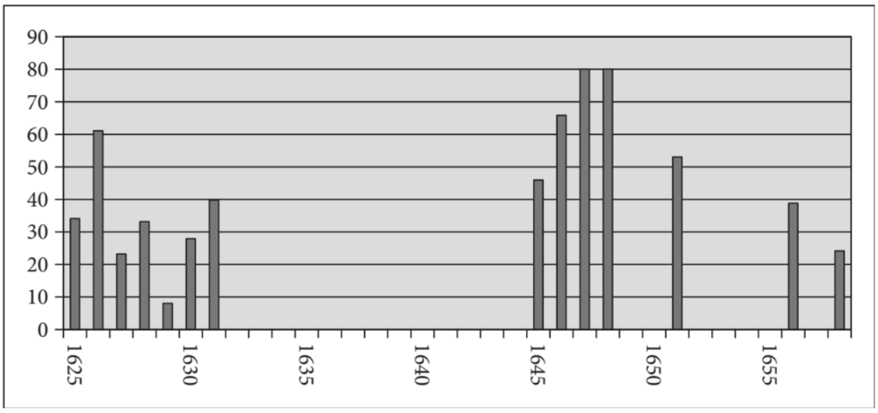

Based on: Oldewelt, *De oudste lastgeldrekeningen*, 5; Wätjen, *Die Niederländer im Mittelmeergebiet*, 406–414; Heeringa (ed.), *Bronnen tot de geschiedenis 1590–1660*, 152.

Figure 2. Number of ships involved in Amsterdam-Mediterranean trade, 1625–1658.[79]

9. Dumrec B (2017) Flottes Publiques, flottes privees a Venise (XII -XV siecles). In : Balard, Michel (ed.) La Mer dans L'Histoire, le Moyen Age, Suffolk, UK: Boydel Press, pp. 138-147.

Summary: Venice started trading with Flanders and England in years 1314 and 1318, respectively. Between 1301-1453, 1924 vessels operated under the Venetian fleet. Between 1443-1456, 128 galleys sailed between Venice and the Orient. (pages: 143-144). In 1499-1500 a marine battle, called “the deplorable battle” emerged in the Peloponnese, between Venice and the Turks with hundreds of vessels from both sides that were engaged (page 144). Further. between 1422-1452 at least 196 sailed between Venice and Syria (page 147).

10. Lopez RS (1951) Majorcans and Genoese on the North Sea Route in the Thirteenth Century. In: Revue belge de philologie et d'histoire, tome 29, fasc. 4, pp. 1163-1179.

“…a number of Genoese notarial documents, the oldest of which go back to 1277 and 1278, have afforded definite proof that Genoese ships reached Flanders and England that early” (based on footnote 3; page 1163). Nevertheless, Genoese at times, are found sailing in foreign bottoms from La Rochelle, Bayonne and other Atlantic seaports of Europe before 1277-1278 (page 1164).”

“As late as the fifteenth century, when trade on the Mediterranean-North Sea route was at its highest mediaeval level, it was not easy to fill to capacity many galleys. The average number of Venetian galleys which undertook that voyage was no more than 14 a year” (based on footnote 1; page 1167).

11. Kirk T (2001) Genoa and Livorno: Sixteenth and Seventeenth-century Commercial Rivalry as a Stimulus to Policy Development. History 86, 3-17.

“Antonio Roccatagliata reports that by early 1592 the price of wheat had fallen from 45 lire per mina to 30, and that, on the single day of 18 January 1593, 130 ships laden with grain entered the port of Genoa” (footnote 13 page 7)

“The seventeenth century was to witness a deep structural change in the nature of the Mediterranean trade. The overwhelming majority of grain shipments made from northern Europe to the Mediterranean region during the famines of the 1590s were sent in northern European ships. As Fernand Braudel and many other historians have pointed out, the previously sporadic presence of English and Dutch ships and merchants in the Mediterranean was intensified during the 1590s.22 Having thus established a presence in the Mediterranean, a great number of northern ships that had arrived carrying foodstuffs remained for relatively long periods, engaging in the rich infra-Mediterranean trades.”

“On the whole, the success of the measures taken in 1654 to increase maritime traffic in Genoa was undeniable; the level of traffic entering the port rose to 1,013,530 cantari* in 1654 and 1,285,700 in 1655, compared to an average of roughly 718,000 cantari for the three preceding years.35 The figure of 985,500 cantari for 1656 would in all probability have been much higher, even surpassing that of the previous two years, if the plague had not broken out in June of that year, bringing port traffic to a halt. There can be no doubt concerning which of the 1654 innovations contributed most to the dramatic increase in port traffic. In 1655 only eight of the 396 merchant ships entering the port took part in the convoy organized by the state, and participation in the convoys diminished during the following years. The republic continued to organize convoys for nearly thirty years, but they were no longer presented as an alternative to the free port for increasing maritime traffic.” *1cantaro =50 kg (approximately).

12. Lopez RS (1964) Market Expansion: The Case of Genoa. The Journal of Economic History 24, 445-464.

This source details the directions of the Genovese shipping: Eastwards westwards and southwards. Although both, Venice and Genova maintained long term naval connections with the levant, the black sea countries and even established a colony in Crimea. However, “Genoa lies farther from the Levant and Africa than her two major medieval rivals, Venice and either Pisa or Pisa's heir, Florence; but she has the best location for trade in the western Mediterranean. Here, Barcelona alone was for some time a serious competitor, and that time was before the fifteenth century. We have seen that the western Mediterranean, not the Levant, was the earliest focus of Genoese trade.” (page 456).

13. Masson P (1967, first published 1896) Histoire du Coerce Français dans le Levant au XVIIIe Siecle, N.Y., Burt Franklin, pp.407 – 417.

This source describes an intensive trade between the Levant and Southern European ports in France (Marseilles, Toulon, Languedoc) established already in the 17th century and onwards (pages 355-502).

14. Van der Wee, H (1990) Structural changes in European long-distance trade, and particularly in the re-export trade from south to north, 1350-1750. In: James D. Tracy (ed.) The Rise of Merchant Empires Long-Distance Trade in the Early-Modern World, 1350-1750. Cambridge, Cambridge University Press, pp. 19-20.

Summary: At the end of the 13th century the first Italian galleys from Venice, Florence and Genoa left the Mediterranean for northwestern Europe, going mainly to Bruges Antwerp and London. During the 14th and 15th centuries this direct maritime that connected between Italy and the North Sea ports became a regular one, replacing to some extent the overland trade via France. The expansion of the north-south maritime trade stimulated commercial activity in ports along the new sea route: the ports of Catalonia, Mallorca and Valencia and of Andalusia, Portugal and the French Atlantic coast benefitted from the galleys moving northward (more details in footnote 8).

15. Cartwright, M. (2016). The Phoenicians Master Mariners. *World History Encyclopedia*. Retrieved from https://www.worldhistory.org/article/897/the-phoenicians---master-mariners/

“The Phoenicians did not have the compass or any other navigational instrument, and so they relied on natural features on coastlines, the stars, and dead-reckoning to guide their way and reach their destination. The most important star to them was the Pole Star of the Ursa Minor constellation and, by way of a compliment to their sea-faring skills, the Greek name for this group was actually *Phoenike* or 'Phoenician'. Some maps of coastal stretches are known to have existed but were unlikely to have been used during a voyage. Rather, navigation was achieved through the position of the stars, sun, landmarks, direction of the winds, and the experience of the captain of tides, currents and winds on the particular route being taken. Close to shore, Herodotus mentions the use of sounding leads to measure the sea depth, and we know that Phoenician ships had a crow's nest for greater visibility.”

16. Horden P, Purcell N (2000). The Corrupting Sea: A Study of Mediterranean History. Blackwell Publishing Ltd.

1) Map 9 in page 128 shows the visibility of land from the sea. 2) Map 12 in page 141 shows two trips from Genoa to the levant in the 14th century. One trip lane was in areas of sight of land for most of the time and the other all the time.
